# Reduced fitness of secondary females in a polygynous species: a 32-yr study of Savannah sparrows

**DOI:** 10.1093/beheco/arae093

**Published:** 2024-11-23

**Authors:** Sarah D Mueller, Nathaniel T Wheelwright, Daniel J Mennill, Amy E M Newman, Stéphanie M Doucet, Joseph B Burant, Sarah L Dobney, Greg W Mitchell, Hayley A Spina, Bradley K Woodworth, D Ryan Norris

**Affiliations:** Department of Integrative Biology, University of Guelph, 50 Stone Rd E, Guelph, ON N1G 1Y2, Canada; Department of Biology, Bowdoin College, 255 Maine St, Brunswick, ME 04011, United States; Department of Integrative Biology, University of Windsor, 401 Sunset Ave, Windsor, ON N9B 3P4, Canada; Department of Integrative Biology, University of Guelph, 50 Stone Rd E, Guelph, ON N1G 1Y2, Canada; Department of Integrative Biology, University of Windsor, 401 Sunset Ave, Windsor, ON N9B 3P4, Canada; Department of Integrative Biology, University of Guelph, 50 Stone Rd E, Guelph, ON N1G 1Y2, Canada; Department of Integrative Biology, University of Windsor, 401 Sunset Ave, Windsor, ON N9B 3P4, Canada; Department of Integrative Biology, University of Guelph, 50 Stone Rd E, Guelph, ON N1G 1Y2, Canada; Department of Integrative Biology, University of Guelph, 50 Stone Rd E, Guelph, ON N1G 1Y2, Canada; Department of Integrative Biology, University of Guelph, 50 Stone Rd E, Guelph, ON N1G 1Y2, Canada; Department of Integrative Biology, University of Guelph, 50 Stone Rd E, Guelph, ON N1G 1Y2, Canada

**Keywords:** fitness, mating systems, multiple mates, parental care, polygyny, reproductive success, Savannah sparrow, sexual conflict

## Abstract

The evolution of mating systems reflects a balance of the often-conflicting interests of males and females. Polygyny, a mating system in which males have multiple mates, presents a fitness benefit to males, but the consequences for females are less clear. Females with polygynous social mates may suffer reduced fitness, especially secondary females who typically receive less male support. We used 32 yr of detailed reproductive data on a population of Savannah sparrows (*Passerculus sandwichensis*) breeding on Kent Island, NB, Canada, to investigate the effects of females’ social mating status on 6 indices of female fitness: survival, clutch size, fledging success, number of fledglings produced per nest and annually, and recruitment of offspring. Secondary females produced fewer fledglings per nest and annually than did monogamous or primary females, and their young were less likely to recruit into the breeding population. Yearling secondary females also had lower survival rates than older secondary females. Combined with higher rates of partial brood loss among secondary females, our results suggest that secondary females are unable to provide enough care to consistently fledge all nestlings in their broods, likely due to reduced male provisioning. Given that the sex ratio of breeders in the population is female-biased, we suggest that polygyny persists despite its fitness costs because some females must mate polygynously to “make the best of a bad situation.” Our study demonstrates the value of detailed, long-term population monitoring data for understanding mating systems and using multiple indices of fitness to analyze the costs of polygyny.

## Teaser

In some species where social monogamy is the predominant mating system, a portion of males may have multiple mates simultaneously (polygyny). Whether females mated to these males experience costs is not clear. Using 32 yr of Savannah sparrow breeding data, we show that secondary females in a polygynous pairing produce fewer fledglings and recruits than primary or monogamous females. Because the adult sex ratio is female-biased, females may have no choice but to mate polygynously.

## Introduction

The evolution of mating systems and mate choice reflects a balance of the often-conflicting interests of males and females (Alatalo et al. 1981; Davies 1989). Polygyny, a mating system in which males have more than one mate, has been proposed to arise either from males’ capacity to monopolize females (Emlen and Oring 1977) or female mate choice (Orians 1969, Santoro et al. 2022). While polygyny presents a potential fitness benefit to males—more mates allow a male to produce more offspring—the consequences for females are less clear (Orians 1969; Altmann et al. 1977; Wheelwright et al. 1992). In polygynous species where males provide some amount of parental care, a male’s attention is divided among multiple mates, with more attention typically paid to early-born offspring from the “primary” female than to later-born offspring from the “secondary” female (Alatalo et al. 1982; Huk and Winkel 2006). In addition to reduced contributions from males, polygynously mated females may also suffer the costs of sharing territory resources with other females, including food and suitable breeding sites (Altmann et al. 1977). Thus, choosing to mate with an already-mated male would appear to be unfavorable for a female.

The predominant explanation for the evolution and maintenance of polygyny is the polygyny threshold model (Orians 1969; Wittenberger 1976), which posits that females choose to mate with an already-mated male if the cost of sharing a mate in terms of divided parental care and territory resources is outweighed by the benefit of a higher quality “breeding situation”—the combination of territory quality, genetic quality of the male, and level of parental care the male provides. Where there are little or no fitness costs of pairing with already-mated males, there is no reason for females to actively avoid mating polygynously, and females may settle and mate at random, without regard to males’ mating status (Lightbody and Weatherhead 1988; Hartley and Shepherd 1995). In cases where the fitness cost of polygyny is low and the quality of the breeding situation is high (e.g. if the male controls a high-quality territory), then mating polygynously could be more beneficial to females than mating monogamously. The polygyny threshold model has received some empirical support (e.g. Pribil and Searcy 2001), but others contend that its assumptions have not been adequately tested (Davies 1989). A central assumption of this model is that females bear some cost of mating polygynously, but this assumption may not hold true in all cases (Wheelwright et al. 1992; Santoro et al. 2022).

Polygynously mated females may suffer a reduction in many aspects of their fitness, which may be especially pronounced for secondary females: females that pair with already-mated males and begin breeding later, who generally receive less male attention than primary females (Alatalo et al. 1982; Huk and Winkel 2006). First, the increased reproductive effort by polygynously mated females could reduce their future survival (Wheelwright et al. 1992; Santoro et al. 2022). Life-history theory predicts a trade-off between the costs of reproductive effort and survival: spending more resources on current reproductive activities can reduce an individual’s ability to perform other maintenance functions, resulting in lower survival (Williams 1966). Because polygynous males divide their attention among multiple females, their mates may be forced to invest more time and resources in parental care to make up for the deficit (Alatalo et al. 1982; Temrin et al. 1996; Santoro et al. 2022). This increased time investment in parental care by polygynously mated females may also have implications for resource acquisition, further exacerbating differences in allocation and survival-reproduction trade-offs (van Noordwijk and de Jong 1986). Additionally, polygynously mated females may produce fewer offspring if they adjust their laying effort according to the available level of male parental care (Schlicht and Kempenaers 2021), effectively optimizing their clutch size for a given breeding scenario (Godfray et al. 1991). Females receiving less assistance from males may also fledge fewer young when male assistance is necessary to successfully raise young to independence (Weatherhead 1979; Alatalo et al. 1982; Huk and Winkel 2006). In birds, males often provide a large proportion of care for fledglings, particularly in double-brooding species where the female begins a second brood while first-brood fledglings are still dependent on parental care (Verhulst and Hut 1996; Green and Cockburn 2001; Wheelwright et al. 2003). Fledglings from the nests of secondary females may receive less attention from males and thus have a lower probability of surviving and recruiting into the breeding population (Lubjuhn et al. 2000; Huk and Winkel 2006).

Empirical studies in birds have shown conflicting results on whether polygyny bears a fitness cost for females, with variation by species, year, whether the study differentiated primary and secondary status of polygynously mated females, and the measures of reproductive success used (e.g. number of hatchlings, fledglings, or grand-offspring; female survival; and current, future, or lifetime reproductive success). A review by Slagsvold and Lifjeld (1994) found that secondary females typically have lower reproductive success compared to monogamous females. A number of other studies have found that decreased parental care from males can lead to lower reproductive success for secondary females (Alatalo et al. 1981, 1982; Johnson et al. 1994; Lubjuhn et al. 2000; Pribil and Searcy 2001; Moreno et al. 2002). Other studies have found little to no reduction in reproductive success or survival for primary or secondary polygynously mated females (Garamszegi et al. 2004; Canal et al. 2021) or costs in some reproductive measures but not others (Both 2002; Huk and Winkel 2006). This variation in results highlights the importance of using multiple indices of fitness, given that it may be easy to miss important costs of mating status by using only one or a few measures (Searcy and Yasukawa 1989; Garamszegi et al. 2004). Relatively few studies, for instance, have investigated the effects of polygyny on recruitment in songbirds, and the majority of these have studied the polyterritorial *Ficedula* flycatchers (collared flycatcher, *F. albicollis*: Garamszegi et al. 2004; pied flycatcher, *F. hypoleuca*: Bensch 1996; Lubjuhn et al. 2000; Both 2002; [Bibr CIT0020][Bibr CIT0010]; blue tit, *Cyanistes caeruleus*: Schlicht and Kempenaers 2021; great reed warbler, *Acrocephalus arundinaceus*: ). Among these investigations, reduced recruitment of young secondary females was only found in pied flycatchers (Lubjuhn et al. 2000; Both 2002; Huk and Winkel 2006; but see Canal et al. 2021). Differentiation between primary and secondary females and longer-term studies that capture year-to-year variation are needed to clarify the effects of polygyny on recruitment.

Here, we use 32 yr of detailed, individual-level reproductive data from a population of Savannah sparrows (*Passerculus sandwichensis*) breeding on Kent Island, New Brunswick, Canada, to investigate the costs to females of mating polygynously using multiple indices of female fitness, both short- and long-term. We use “monogamy” and “polygyny” to refer to social mating systems, in which males have one (monogamous) or multiple social mates (polygynous). Polygyny is common in many populations of Savannah sparrows (Welsh 1975; Wheelwright and Rising 2020), including the Kent Island population (Wheelwright et al. 1992), which has a consistently female-biased sex ratio of breeders. The frequency of polygyny among males averages 23% but varies from 0-55% across years (this study). A previous study on the Kent Island population found little effect of polygyny on indices of female fitness, with the exception of one year where overwinter survival of polygynous females and recruitment of their offspring were lower compared to monogamous females (Wheelwright et al. 1992). However, for this study, only 5 yr of data were available, and the authors did not quantify differences between primary and secondary polygynously mated females (Wheelwright et al. 1992). In the Kent Island population, the amount of parental care provided by polygynous males to nests of primary females during the nestling stage does not differ from that of monogamous males but tends to be lower to nests of secondary females (Wheelwright et al. 1992; Freeman-Gallant 1998). Females typically provide the majority of food deliveries to nestlings at both monogamous and polygynous nests, with males bringing, on average, less than 30% of food deliveries (range 0% to 75%), and females make up for lack of male assistance by increasing their rate of food deliveries (Wheelwright et al. 1992; Freeman-Gallant 1998). Females are often able to raise at least some nestlings without male assistance but may suffer reduced fledging success (Welsh 1975; Weatherhead 1979; Wheelwright et al. 1992). Here, we investigate the effects of females’ mating status—paired monogamously, or as the primary or secondary mate of a polygynous male—and other intrinsic and extrinsic factors on 6 indices of female fitness: survival of females, clutch size, fledging success, number of fledglings produced per nest, annual number of fledglings produced, and recruitment of offspring. Table 1 provides a detailed list of hypotheses and predictions that address how we expect each of these reproductive success metrics to be influenced by the mating status of a female.

**Table 1.** Hypotheses and predictions for the effect of mating status (monogamous, primary or secondary polygynous) on female fitness indices.

Fitness Index	Hypothesis	Prediction
Survival of female	2° females receive less help from males, requiring them to devote more energy to reproduction, resulting in lower survival	2° females will have lower survival than either monogamous or 1° females
Clutch size	Females lay the number of eggs that results in the maximum number of offspring they can fledge; females that receive more assistance with parental care from males (monogamous, 1°) can successfully rear more young, so lay larger clutches	Monogamous and 1° females will lay larger clutches than 2° females
Fledging success	Secondary (2°) females receive less help feeding nestlings from males than monogamous or primary (1° females) and may not be able to successfully rear any nestlings to fledging alone	2° females will have lower fledging success than either monogamous or 1° females
Number of fledglings per nest	2° females receive less help feeding nestlings from males and cannot successfully feed and fledge as many young	2° females will produce fewer fledglings per nest than monogamous and 1° females
Number of fledglings per year	2° females receive less help from males and may have decreased fledging success, fewer nest attempts per year, or fledge fewer young per nest	2° females will produce fewer fledglings per year than monogamous and 1° females
Recruitment of offspring	Offspring of 2° females receive less care from males as both nestlings and fledglings	Fledglings from nests of 2° females will be less likely to recruit than fledglings from nests of monogamous and 1° females

## Methods

### Study system and field methods

**Fig. 1.**
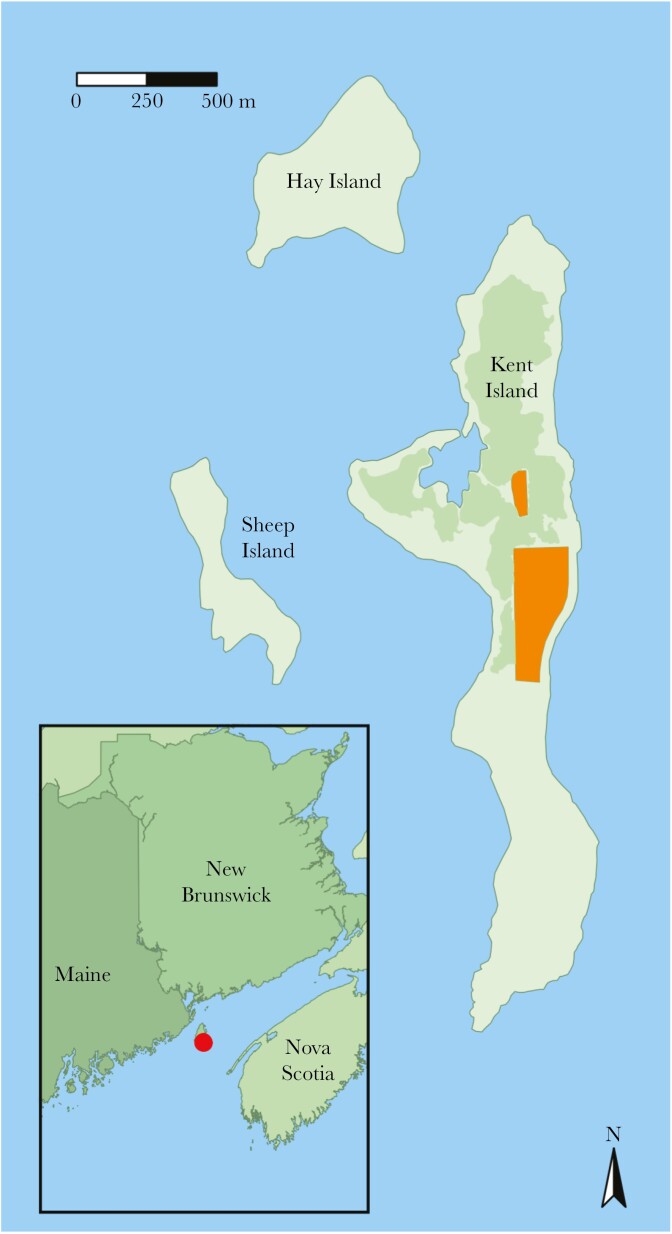
Map of the long-term Savannah sparrow study site on Kent Island, NB, CA (44.58° N, 66.75° W) and the neighboring islands, Hay and Sheep, located off the coast of Maine in the Bay of Fundy (inset). Annual monitoring of Savannah sparrows occurs within the shaded study area in the center of the island, which consists of 2 areas of open field about 100 m apart, separated by mixed coniferous forest.

Savannah sparrows are migratory songbirds that breed in grasslands and open habitats across North America and winter in the southern United States, Mexico, and parts of Central America (Wheelwright and Rising 2020). Our study population breeds on Kent Island, NB, Canada (44.58° N, 66.75° W), an approximately 100 ha island in the Bay of Fundy. The biology of sparrows breeding in a ~10 ha study area in the center of the island has been studied annually since 1987 (excluding 2005 to 2007 and 2020; Fig. 1). Annual monitoring between late May and late July consists of mapping territories, searching for and monitoring all nests, color banding all new members of the population and nestlings, and resighting banded individuals. All breeding adults are given a unique combination of a United States Fish and Wildlife Service/Canadian Wildlife Service aluminum leg band and 3 colored plastic leg bands. The sex of adults was determined at capture by the presence of a cloacal protuberance in males or a brood patch in females and confirmed by behavior: only males sing and only females build and incubate at nests (Wheelwright and Rising 2020). The ages of breeding adults were known with certainty if they were banded as nestlings or juveniles in preceding years. We categorized all breeding adults as either yearlings breeding for the first time (second-year, “SY”) or older birds (after second-year, “ASY”). We assumed that birds that were first captured as unbanded adults were yearlings dispersing into the study site due to the very small breeding dispersal distances in this population; adults that have bred before disperse only 20 to 30 m on average from previous years’ nest sites (Wheelwright and Mauck 1998; Hensel et al. 2022). Most birds first captured as unbanded adults could be confirmed as yearlings using feather shape and wear (Pyle 1997). To account for the negative effects of conspecific density on fitness (Woodworth et al. 2017), we calculated annual population density (birds/ha) as the number of breeding adults on the main study site each year, divided by 10.7 ha for the area of the study site.

Nests, which were primarily found during egg laying or incubation, were monitored every other day to determine final clutch size, number of nestlings and fledglings, hatching and fledge dates, and nest fate. Nestlings were banded and weighed on the seventh day after hatching. On Kent Island, female Savannah sparrows typically lay 4 to 5 eggs per clutch and can successfully fledge up to 2 broods per year (on average, ~30% of females attempt a second brood, Woodworth et al. 2017). Females may renest as many as 5 times if previous nest attempts fail (Dixon 1978). The incubation period lasts 12 d on average, and young typically fledge 9 to 11 d after hatching (Dixon 1978). For nests where eggs hatched, we estimated the date on which the first egg was laid (hereafter “first egg date”) as the hatch date minus 12 (number of days of incubation) minus one less than the clutch size (females begin incubating after laying the penultimate egg; Burant et al. 2022). For nests where no eggs hatched, we estimated the first egg date by first calculating the average difference between the date nests were found and their hatch dates for all nests in a given year. We estimated hatch dates for these nests by adding these yearly average differences to the date the nest was found (Woodworth et al. 2017). We then estimated the first egg date for these nests as described above. Females build nests and incubate eggs alone, without male assistance, though males may help by watching for predators and giving alarm calls to warn incubating or foraging females (Wheelwright et al. 1992; Wheelwright and Rising 2020). Both parents feed nestlings, though females usually provide more food (Welsh 1975; Wheelwright et al. 1992). After fledging, the parents divide the brood, and each cares for 1 to 3 fledglings; females may begin a second clutch while still feeding first-brood fledglings.

The social mate of each female was determined by noting which male’s territory her nest fell within, as well as which male was observed copulating, mate-guarding, and closely associating with the female and which male later fed nestlings at her nest. In most cases, each criterion produced the same assignment of a social mate. In some cases, however, a female was initially matched with a male that was observed affiliating with her but later matched with a different male if he was observed closely associating with her and feeding nestlings at her nest. When assignments of social mates conflicted, the identity of the male feeding nestlings weighed more heavily than other criteria. Females were defined as monogamous if their assigned social male had no other concurrent social mates and polygynous if their assigned social male had at least one other concurrent social mate. Polygynous females were designated as primary if they were the first-laying females mated to a polygynous male and secondary if they were not the first-laying females (thus “secondary” also includes tertiary and lower-level females). Hereafter, we will refer to monogamously mated females as “monogamous females” (abbreviated as “MO”), the primary mates of polygynous males as “primary females” (“PG1”), and the secondary mates of polygynous males as “secondary females” (“PG2”).

Our nest dataset spanned from 1987 to 2023, excluding years with incomplete monitoring (2005 to 2007, 2020) and years where mating status was not recorded (2008 to 2010), and included only nests where mating status was known with certainty (*n* = 2,683 nests). Here, when we refer to “survival” and “recruitment,” we are reporting return rates. Due to strong natal and breeding philopatry in this population, we assumed that birds that were not resighted in any following year had died (Wheelwright et al. 1992; Wheelwright and Mauck 1998). Return rates average 45% for adults, 11% for nestlings, and 26% for fledglings (Wheelwright and Schultz 1994); these would be high rates for similarly sized migratory passerines if any significant proportion of birds also dispersed (adult survival: Martin 1995; juvenile survival: Beauchamp 2023). Additionally, few birds born or breeding within the study area are found during annual censuses of Kent Island outside the study area and on the surrounding islands (DRN, BKW, JBB, SDM, unpublished data). Within the study area, detection probability was high: out of 11,175 individuals in our long-term study from 1987 to 2022, only 113 individuals were missed in one or a few years between years where they were detected (~1% of individuals). Therefore, we chose not to use capture–recapture analysis for survival or recruitment.

### Statistical analysis

The effect of female mating status on the indices of female fitness was tested using generalized linear mixed models (GLMMs). Each analysis also accounted for the effects of other intrinsic and extrinsic factors hypothesized to influence female fitness (e.g. brood size, ordinal dates, age of females, and population density). All continuous predictors were grand-mean centered, unless otherwise noted, and models were fit using sum-to-zero coding for categorical predictors. All analyses were conducted using the R statistical environment (v. 4.2.3; R Core Team 2023). Models were fit using the package glmmTMB (v. 1.1.7; Brooks et al. 2017), model diagnostics checked using DHARMa (v. 0.4.6; Hartig 2022), and estimated marginal means generated using emmeans (v. 1.8.5; Lenth 2023). For each response variable, we fit a full model with all predictor variables and select interactions suspected to be biologically important. We then removed interaction and higher-order terms that were not significant (*P* < 0.05) to improve the interpretability of the main effects of the terms involved and fit these models. We present results from these reduced models, with non-significant terms removed (see Appendix 1 for results of the full models). Pairwise comparisons between levels of categorical variables were carried out using the emmeans function and estimated trends for interactions between a categorical variable and a continuous covariate (e.g. clutch number and date) were carried out using the emtrends function, both in the package emmeans (v. 1.8.5; Lenth 2023). The following is a description of each of the 6 models focused on the different indices of female fitness.

#### Female survival.

Female survival was defined as whether or not a female that bred in year *t* returned (i.e. nested or was recaptured) in year *t + *1 and was modeled using logistic regression (GLMM, binomial distribution, logit link) with random effects for year and female ID. The full model included female mating status in year *t*, the number of young fledged in year *t*, population density in year *t*, female age in year *t*, and all possible 2-way interactions. If a female was recorded as both monogamously and polygynously mated in different nest attempts in a given year, she was considered to be polygynously mated that year. We excluded from this analysis years for which no monitoring took place the subsequent year (2004 to 2006 and 2019) or when insufficient time had elapsed to detect returning females (2023), which addressed issues associated with right-censored data arising from incomplete observations. For the reduced model, we removed all interactions except female status × female age (full model: *P* = 0.01; other interactions *P* > 0.3; Appendix 1: Table S1).

#### Clutch size.

Clutch size, the number of eggs laid per nest, was modeled using a Generalized Poisson rate GLMM (log link) with an offset of the log of the maximum number of eggs per clutch, which is 5 eggs in this population. Female ID and year were included as random effects. The full model included female mating status, population density, female age, clutch number (first or second), first egg day (group-mean centered by clutch number and female status), and interactions between female status and population density, female status and clutch number, population density and female age, and clutch number and first egg date. For this analysis, we excluded nests with experimentally manipulated clutch sizes, and nests with clutch sizes of 0 or 1, as these were almost certainly incomplete clutches that were depredated or abandoned before females finished laying. For the reduced model, we removed all interactions except clutch number × first egg date (full model: *P* < 0.001; other interactions *P* > 0.4; Appendix 1: Table S2).

#### Fledging success.

Fledging success was defined as whether or not a nest survived from hatching to fledging and was modeled using logistic regression (GLMM, binomial distribution, logit link) with random effects for year and female ID. The full model included female mating status, brood size (categorical, with values 1 to 2, 3, 4, or 5 nestlings), a linear and quadratic term for first egg date (group-mean centered by female status), female age, population density, and interactions between female status and brood size, female age, and population density; brood size and female age; and brood size and population density. For this analysis, we excluded nests in which brood size or nest survival was experimentally manipulated (e.g. clutch enlargement or predator exclosures) and nests that never hatched or had unknown fates. For the reduced model, we removed all interactions (full model: all interactions *P* > 0.06; Appendix 1: Table S3).

#### Fledglings per nest.

The number of fledglings produced from a single nest was modeled using a zero-inflated generalized Poisson GLMM (log link, intercept-only zero-inflation term) with random effects for year and female ID. The full model included female mating status, female age, clutch number, population density, first egg day (group-mean centered by clutch number and female status), and interactions between female status and female age, first egg day, population density, and clutch number; female age and population density; and first egg day and clutch number. For this analysis, we excluded nests for which brood size or nest survival were experimentally manipulated (e.g. clutch enlargement or predator exclosures) and nests that never hatched or had unknown fates. For the reduced model, we removed all interactions except clutch number × first egg date (full model: *P* < 0.001; other interactions *P* > 0.4; Appendix 1: Table S4).

#### Fledglings per year.

The number of fledglings a female produced in a given year across all nest attempts was modeled using a generalized Poisson GLMM (log link) with random effects for year and female ID. The full model included female mating status, number of successful nest attempts (as a factor variable, 1 or 2 nests), population density, female age, date of breeding commencement (the first egg day of a female’s first nest attempt, group-mean centered by female status), and interactions between female mating status and population density, female mating status and female age, and population density and female age. We excluded females that produced no fledglings in a given year. There was no significant difference in the proportion of females that fledged no nests between the different female mating status levels (chi-square goodness-of-fit test, χ^2^ = 3.36, *P* = 0.186). We also excluded females that had at least one nest with experimentally manipulated clutch or brood size or fledging success (e.g. nests with predator exclosures). For the reduced model, we removed all interactions (full model: all interactions *P* > 0.3; Appendix 1: Table S5).

#### Recruitment of young.

Recruitment of a female’s young from fledging to their first breeding season, defined as whether or not a fledgling was recorded as a breeder or was recaptured in subsequent years, was modeled using logistic regression (GLMM, binomial distribution, logit link) with random effects for year and female ID. The full model included the mating status of the fledgling’s mother, population density, the combination of the parents’ ages (i.e. both parents ASY, both parents SY, or one parent ASY and one SY), fledge date (group-mean centered by female status), the fledglings’ weight as a nestling, and interactions between female status and population density, female status and parents’ ages, population density and parents’ ages, and population density and fledge date. Some fledglings were missing fledge dates; these missing dates were estimated with a minimal error by adding 9 d to the hatch day (mean difference between hatch and fledge day ± SE: 9.18 ± 0.01 d). We excluded from this analysis years for which no monitoring took place the subsequent year (i.e. 2004 and 2019) or when the insufficient time had elapsed to detect returning recruits (i.e. 2023). For the reduced model, we removed all interactions (full model: all interactions *P* > 0.1; Appendix 1: Table S6).

## Results

The full nest dataset from 1987 to 2023 included 2,683 nests, of which 1,898 were associated with monogamous pairs, 377 were associated with the primary female of a polygynous male, and 408 were associated with the secondary female of a polygynous male. The proportion of females in each mating status category varied widely between years (*n* = 30 years), with, on average, 67% of females being monogamously mated (range 28% to 100%), 14% of females being the primary female of a polygynous male (range 0% to 34%), and 19% of females being the secondary female of a polygynous male (range 0% to 46%).

### Female survival.

**Fig. 2.**
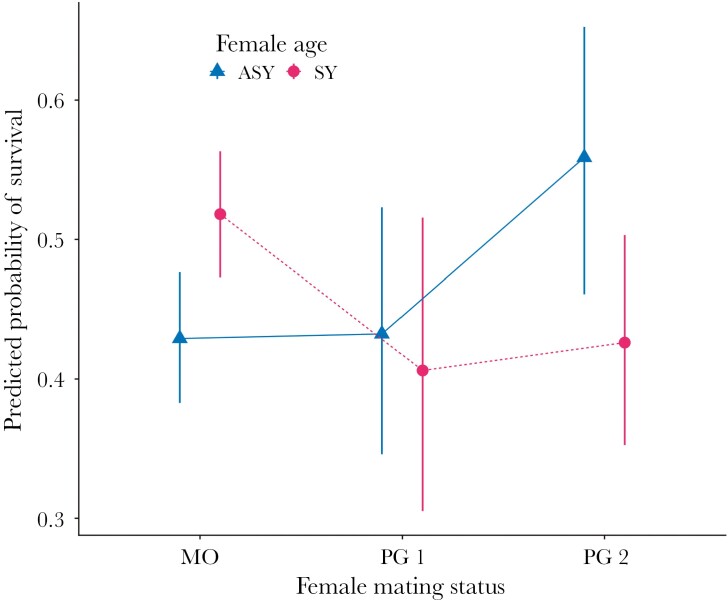
Effect of age and mating status on survival of female Savannah sparrows. Among secondary females, older individuals (ASY) were significantly more likely to survive to a subsequent year than yearlings (SY), while among monogamous females, older individuals were less likely to survive than yearlings. There was no difference in survival among primary females by age. Model predictions are estimated marginal means with 95% CIs from the final model for female survival, holding other continuous predictors at their mean values and averaging across levels of other categorical predictors (GLMM; model predictions generated using the emmip function in the R package emmeans).

The final model for the survival of females to the following breeding season included female mating status, the number of fledglings she produced in a year, population density, female age, and the interaction of female mating status and age (*n* = 1428 female-year combinations; 759 did not return, 669 did return; GLMM, binary distribution, logit link; Table 2). Over half of females (58%) appeared in only one year in the dataset; 26% appeared in 2 yr, and the remainder appeared in 3 to 7 y (3 to 4 yr: 13%, 5 to 7 y: 3%). The interactive effect of female mating status and female age on female survival was significant (GLMM, χ^2^ = 10.50, *df* = 2, *P* = 0.01, Fig. 2). The probability of survival was significantly higher for older (ASY) secondary females than for younger (SY) secondary females (pairwise comparison, *z* = 2.09, *P* = 0.04) but lower for older monogamous females than younger ones (*z* = −2.65, *P* = 0.01). There was no difference in survival among primary females by age (*z* = 0.37, *P* = 0.71). The probability of a female returning in a subsequent breeding season increased with the number of fledglings produced in the preceding year. For each additional fledgling produced, a female’s predicted odds of returning in a subsequent year increased by 12.1% (Table 2).

**Table 2.** The effects of female mating status, the annual number of fledglings produced, population density, female age, and the interaction of female age and mating status on female survival in Savannah sparrows.

Fixed effects	Estimate (β)	SE	95%CI	*z*-value	*P*-value
(Intercept)	−0.616	0.127	(−0.864, −0.367)	−4.85	**<0.001**
Female status (MO)^a^	0.048	0.078	(−0.106, 0.201)	0.61	0.541
Female status (PG1)^b^	−0.172	0.109	(−0.385, 0.041)	−1.58	0.114
Number of fledglings	0.115	0.026	(0.064, 0.165)	4.46	**<0.001**
Population density	0.003	0.031	(−0.057, 0.064)	0.11	0.914
Female age (ASY)^c^	0.047	0.069	(−0.088, 0.183)	0.68	0.495
Female status (MO) × female age (ASY)	−0.226	0.078	(−0.379, −0.074)	−2.91	**0.004**
Female status (PG1) × female age (ASY)	0.007	0.108	(−0.205, 0.218)	0.06	0.951
**Random effects**	**Variance (σ** ^**2**^ **)**	**SD**			
Year	0.003	0.051			
Female ID	<0.001	<0.001			

^a^Monogamous; ^b^Primary polygynous; ^c^After second-year.

Values in bold indicate significant differences. Sample sizes: years = 27, unique females = 840, female-year combinations = 1,428.

### Clutch size.

**Fig. 3.**
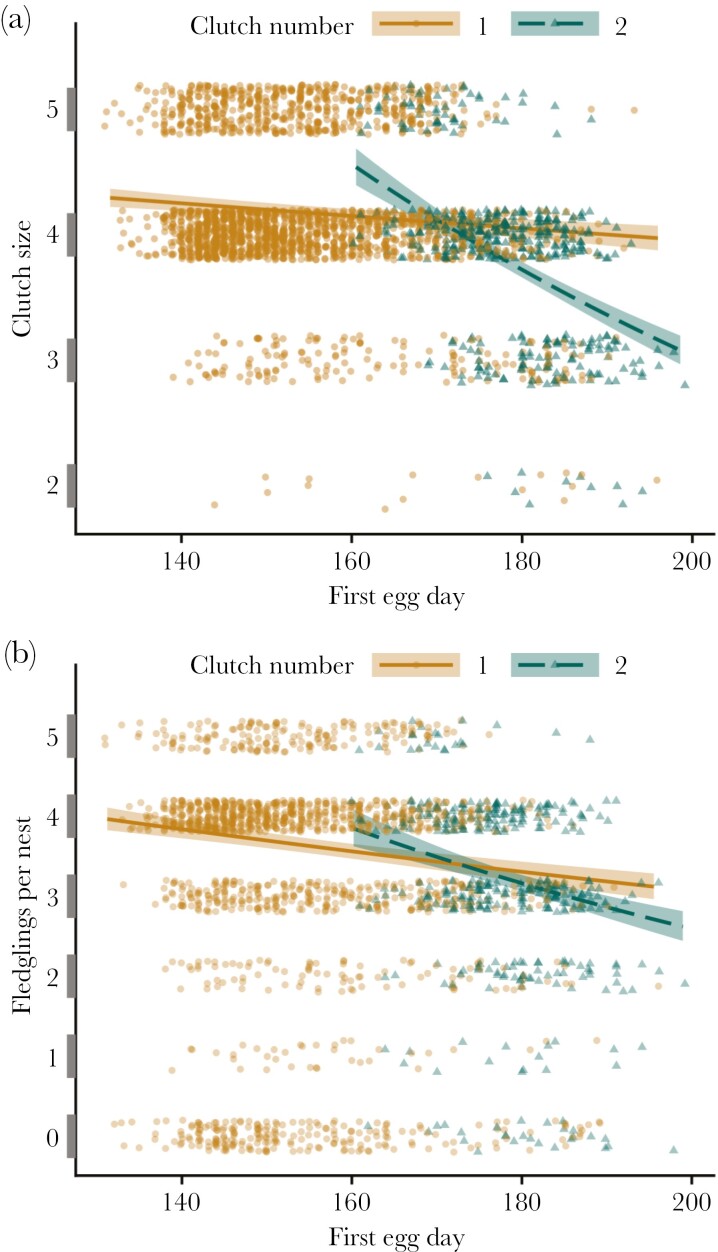
Effect of Savannah sparrow clutch number and ordinal first egg date on (a) clutch size and (b) number of fledglings per nest. Clutch size and the number of fledglings per nest declined with first egg day across both clutch numbers but declined more steeply for second clutches than for first clutches. Points are clutch size (a) or number of fledglings per nest (b) plotted versus first egg date and are jittered around the discrete response variable values. Lines are model predictions with 95% CI from the final models for clutch size and number of fledglings per nest, holding other continuous predictors at their mean values and averaging across levels of other categorical predictors (GLMMs; model predictions generated using the emmip function in the R package emmeans).

The final model for clutch size included female mating status, population density, clutch number, female age, first egg date, and the interaction between clutch number and first egg date (*n* = 2,579 nests; GLMM, generalized Poisson rate distribution, log link; Appendix 2: Table S1). Female mating status had no significant effect on clutch size (GLMM; χ^2^ = 3.41, *df* = 2, *P* = 0.18). Yearling females laid clutches that were, on average, 0.15 eggs smaller than the clutches of older females (SY: 3.91 ± 0.03 eggs, ASY: 4.06 ± 0.03 eggs; GLMM, β_ASY_ = 0.02 ± 0.003, *z* = 7.01, *P *< 0.001). Clutch size decreased with the first egg date for both the first and second clutches but more steeply for the second clutches (GLMM, β = 0.004 ± 0.0004, *z* = 10.54, *P *< 0.001, Fig. 3a): for first clutches, the predicted clutch size decreased by 0.1% per day (estimated trend: −0.0012, 95% CI −0.0017 to −0.0007), while for second clutches, the predicted clutch size decreased by 1% per day (estimated trend: −0.010, 95% CI −0.012 to −0.009).

### Fledging success.

 The final model for fledging success included fixed effects of female mating status, brood size, female age, population density, and linear and quadratic terms for the first egg day (*n* = 1,727 nests; 1,508 had at least one nestling that fledged, 219 did not have any nestlings that fledged; GLMM, binary distribution, logit link; Appendix 2: Table S2). Female mating status did not have a significant effect on the probability of young fledging from a nest (GLMM; χ^2^ = 2.27, df = 2, *P* = 0.32). Fledging success differed by brood size (GLMM, χ^2^ = 8.31, df = 3, *P* = 0.04); broods of 4 nestlings were significantly more likely to fledge than broods of 1-2 nestlings (probability of fledging success ± SE: 1 to 2 nestlings = 0.84 ± 0.05, 4 nestlings = 0.93 ± 0.01; pairwise comparisons, *z* = −2.71, *P* = 0.03). No other pairs of brood sizes were significantly different (*P* ≥ 0.2). Fledging success was lower for yearling females than for older females (GLMM, probability of fledging success ± SE: SY = 0.88 ± 0.02, ASY = 0.92 ± 0.02; β_ASY_ = 0.21 ± 0.08, *z *= 2.49, *P *= 0.01). The probability of fledging success was highest for nests with first egg dates in the middle of the season (early June to mid-July) and lower for nests with early (late May) or late (late July) first egg dates (GLMM; linear date term: β = 0.04 ± 0.01, *z* = 5.97, *P* = < 0.001; quadratic date term: β = −0.001 ± 0.0004, *z* = −2.17, *P *= 0.03).

### Fledglings per nest.

**Fig. 4.**
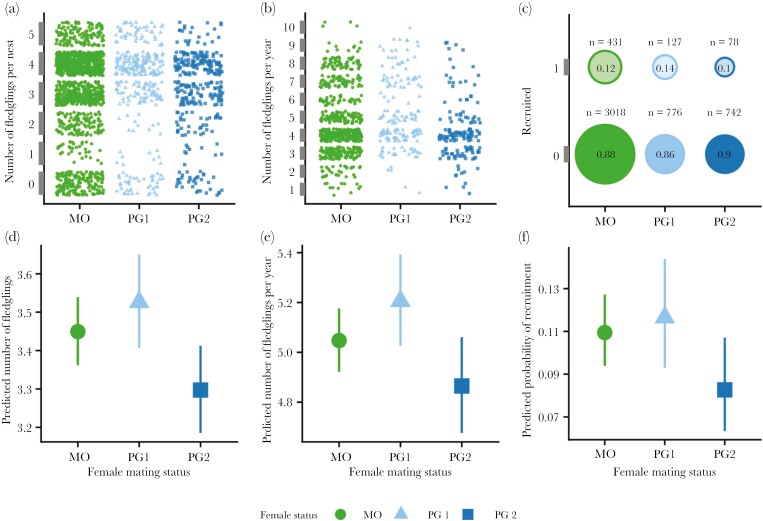
Effect of female Savannah sparrow mating status on number of fledglings per nest (a,d), number of fledglings per year (b,e), and recruitment (c,f), with data on the top row (a–c) and model predictions on the bottom row (d-f). Secondary females produced fewer fledglings per nest and per year than did monogamous and primary females, and the young of secondary females were less likely to recruit. Model predictions are estimated marginal means with 95% CI from the final models for fledglings produced per nest and per year and for recruitment, holding other continuous predictors at their mean values and averaging across levels of other categorical predictors (GLMMs; model predictions generated using the emmip function in the R package emmeans).

The final model for the number of fledglings produced per nest included female mating status, population density, clutch number, female age, first egg date, and a clutch number × first egg date interaction term (*n* = 1,982 nests; GLMM, zero-inflated generalized Poisson distribution, log link; Table 3). Female mating status significantly affected the number of fledglings per nest (GLMM; χ^2^ = 14.22, *df* = 2, *P *< 0.001; Fig. 4a,d). On average, secondary females produced ~0.2 fewer young per nest than did primary or monogamous females (MO: 3.45 ± 0.05 fledglings, PG1: 3.53 ± 0.06 fledglings, PG2: 3.3 ± 0.06 fledglings; pairwise comparison, MO-PG2: *z* = 3.02, *P* = 0.007, PG1-PG2: *z *= 3.65, *P* < 0.001), whereas there was no significant difference between monogamous and primary females (pairwise comparison, *z *= −1.48, *P* = 0.30). Yearling females produced, on average, 0.08 fewer fledglings than older females (SY: 3.38 ± 0.05 fledglings, ASY: 3.46 ± 0.05 fledglings; Table 3). The number of fledglings produced per nest decreased more steeply with the first egg date within second clutches than within first clutches (Table 3, Fig. 3b): for first clutches, the predicted number of fledglings decreased by 0.4% per day (estimated trend: −0.004, 95% CI: −0.005 to −0.003), while for second clutches, the predicted number of fledglings decreased by 1% per day (estimated trend: −0.010, 95% CI: −0.013 to −0.007).

**Table 3.** The effects of female mating status, female age, population density, first egg date, clutch number, and the interaction of clutch number and first egg date on number of fledglings per nest in Savannah sparrows.

Fixed effects	Estimate (β)	SE	95%CI	*z*-value	*P*-value
(Intercept)	1.231	0.013	(1.205, 1.257)	92.40	**<0.001**
Female status (MO)^a^	0.008	0.008	(−0.008, 0.023)	0.99	0.323
Female status (PG1)^b^	0.030	0.010	(0.010, 0.049)	2.98	**0.003**
Female age (ASY)^c^	0.012	0.005	(0.002, 0.022)	2.28	**0.023**
Population density	−0.002	0.007	(−0.015, 0.011)	−0.34	0.737
First egg date	−0.007	0.001	(−0.008, −0.005)	−8.46	**<0.001**
Clutch number (1)	0.059	0.006	(0.046, 0.071)	9.50	**<0.001**
Clutch number × first egg date	0.003	0.001	(0.002, 0.005)	3.87	**<0.001**
**Zero-inflation parameter**					
(Intercept)	−1.938	0.068	(−2.071, −1.805)	−28.58	**<0.001**
**Random effects**	**Variance (σ** ^**2**^ **)**	**SD**			
Year	0.003	0.057			
Female ID	0.003	0.052			
**Dispersion** **parameter** for generalized Poisson family: 0.175					

^a^Monogamous; ^b^Primary polygynous; ^c^After second-year.

Values in bold indicate significant differences. Sample sizes: years = 30, unique females = 875, nests = 1,981.

### Fledglings per year.

The final model for the total number of fledglings produced across all nest attempts by a female in a given year included female mating status, number of successful nest attempts, population density, breeding commencement date, and female age (*n* = 987 female-year combinations; GLMM, generalized Poisson distribution, log link; Table 4). Female mating status significantly affected the number of fledglings produced by a female in a year (GLMM; χ^2^ = 9.51, *df* = 2, *P* = 0.01; Fig. 4b,e). Secondary females produced on average 0.35 fewer fledglings in a year than primary females; there was no significant difference between monogamous females and either primary or secondary females (PG2: 4.86 ± 0.10 fledglings, PG1: 5.21 ± 0.09 fledglings, MO: 5.05 ± 0.06 fledglings; pairwise comparisons, PG1-PG2: *z* = 3.08, *P* = 0.01, MO-PG1: *z *= −1.83, *P* = 0.16, MO-PG2: *z *= 1.94, *P* = 0.13). The number of successful nest attempts was a strong predictor of the number of fledglings per year, with females that had 2 successful nests producing on average almost double the number of fledglings as females that had only one successful nest (one nest: 3.72 ± 0.05 fledglings, 2 nests: 6.82 ± 0.11 fledglings; Table 4). The predicted number of fledglings per year decreased with the breeding commencement date by 0.5% per day so that females that began breeding later in the season fledged fewer young on average than females that began breeding earlier (Table 4). Older females produced 0.13 more fledglings per year, on average, than did younger females (ASY: 5.10 ± 0.07 fledglings, SY: 4.97 ± 0.07 fledglings; Table 4).

**Table 4.** The effects of female mating status, number of successful nests, population density, breeding start date (first egg date of first nest attempt), and female age on the total number of fledglings per year in Savannah sparrows.

Fixed effects	Estimate (β)	SE	95%CI	*z*-value	*P*-value
(Intercept)	1.617	0.013	(1.591, 1.643)	123.08	**<0.001**
Female status (MO)^a^	0.002	0.009	(−0.017, 0.020)	0.21	0.836
Female status (PG1)^b^	0.033	0.011	(0.010, 0.055)	2.87	**0.004**
Number of nests (1)	−0.302	0.007	(−0.316, −0.289)	−43.62	**<0.001**
Population density	−0.002	0.006	(−0.014, 0.010)	−0.31	0.755
Breeding start date	−0.005	0.001	(−0.008, −0.002)	−3.44	**0.001**
Female age (ASY)^c^	0.013	0.007	(0.0, 0.026)	1.95	**0.051**
**Random effects**	**Variance (σ** ^**2**^ **)**	**SD**			
Year	0.003	0.050			
Female ID	0.004	0.061			
**Dispersion parameter** for generalized Poisson family: 0.172					

^a^Monogamous; ^b^Primary polygynous; ^c^After second-year.

Values in bold indicate significant differences. Sample sizes: years = 27, unique females = 611, unique female-year combinations = 987.

### Recruitment.

 The final model for recruitment of a fledgling to the next breeding season included the mother’s mating status, population density, parents’ combined ages, fledge date, and nestling weight (*n* = 5,172 fledglings, 4,536 did not return, 636 did return; GLMM, binary distribution, logit link; Table 5). Mother’s mating status significantly affected a fledgling’s probability of recruitment (GLMM; χ^2^ = 5.95, *df* = 2, *P* = 0.05; Fig. 4c, f). Fledglings from the nests of secondary females tended to be less likely to recruit than fledglings from nests of monogamous or primary females (MO: 0.11 ± 0.01, PG1: 0.12 ± 0.01, PG2: 0.08 ± 0.01; pairwise comparison, MO-PG2: *z* = 2.19, *P* = 0.07; PG1-PG2: *z* = 2.29, *P* = 0.06). There was no significant difference between young monogamous and primary females (pairwise comparison, MO-PG1: z = −0.57, *P* = 0.84). Parents’ ages also significantly affected the probability of recruitment (GLMM, χ^2^ = 6.85, *df *= 2, *P *= 0.03). Young from pairs with 2 older parents tended to be more likely to recruit (both ASY: 0.12 ± 0.01) than young pairs with at least one younger parent (ASY/SY: 0.09 ± 0.01, both SY: 0.10 ± 0.01; pairwise comparisons, both ASY-ASY/SY: *z* = 2.11, *P* = 0.09; both ASY-both SY: *z* = 2.28, *P* = 0.06; ASY/SY-both SY: *z* = 0.03, *P* = 0.95). The predicted odds of a fledgling recruiting tended to decrease by 7% for every one bird per ha increase in population density (Table 5) and decreased by 2% for each day later in the season (Table 5). Nestling weight positively affected recruitment, such that a 1 g increase in weight increased the predicted odds of recruitment by 10% (Table 5).

**Table 5.** The effects of female mating status, population density, parents’ combined ages, fledge day, and mass as a nestling on recruitment of fledgling Savannah sparrows.

Fixed effects	Estimate (β)	SE	95%CI	*z*-value	*P*-value
(Intercept)	−2.177	0.090	(−2.353, −2.000)	−24.18	**<0.001**
Female status (MO)^a^	0.080	0.069	(−0.056, 0.216)	1.16	0.248
Female status (PG1)^b^	0.151	0.086	(−0.017, 0.318)	1.76	0.079
Population density	−0.078	0.041	(−0.157, 0.002)	−1.92	0.056
Age (both parents ASY)^c^	0.166	0.064	(0.042, 0.291)	2.62	**0.009**
Age (both parents SY)^d^	−0.065	0.067	(−0.197, 0.067)	−0.96	0.335
Fledge day	−0.019	0.003	(−0.025, −0.012)	−5.37	**<0.001**
Mass	0.097	0.024	(0.050, 0.144)	4.01	**<0.001**
**Random effects**	**Variance (σ** ^**2**^ **)**	**SD**			
Year	0.082	0.287			
Female ID	0.209	0.457			

^a^Monogamous; ^b^Primary polygynous; ^c^After second-year; ^d^Second-year.

Values in bold indicate significant differences. Sample sizes: years = 27, unique females = 706, number of fledglings = 5,151.

## Discussion

Our results provide a comprehensive picture of the costs of polygyny in a migratory passerine. Female mating status significantly affected the number of fledglings produced per nest, the total number of fledglings produced by a female in a year, and the recruitment of young to the following breeding season. In all cases, secondary females suffered reduced success relative to monogamous and primary females. Additionally, together with female age, mating status influenced the survival of females to subsequent years, with yearling secondary females being less likely to survive than older secondary females. These results support the hypothesis that secondary females have reduced reproductive success in terms of the number of young they can produce and the proportion of their young that survive to breeding age, potentially due to reduced male care for the nestlings and fledglings of secondary females. In the other fitness indices examined—fledging success and clutch size—we found no effect of female mating status, suggesting that females do not appear to adjust their clutch size in response to their mating status and that fledging success is influenced by other factors such as female age and the timing of breeding.

Taken together, our results provide evidence of the specific mechanism by which the breeding success of secondary females is reduced relative to primary and monogamous females. Although secondary females had similar clutch sizes and fledging success compared to primary and monogamous females, they still produced fewer fledglings. This appears to be through a combination of lower hatching success (the proportion of eggs that hatched out of the number laid, excluding depredated nests) and higher rates of partial brood loss, in which some but not all nestlings fledge. Secondary females had lower hatching success and lost more nestlings in their successful nests than did primary females (Table 6); monogamous females also had low hatching success, but this was compensated for by lower numbers of nestlings lost per nest. Female Savannah sparrows typically increase their rates of food deliveries to make up for reduced male assistance (Weatherhead 1979; Wheelwright et al. 1992). However, unassisted females still often raise nestlings that are in worse condition and may not be able to successfully fledge the entire brood alone (Weatherhead 1979; Smith et al. 1982). Our results suggest that secondary females are still able to fledge at least some nestlings but are likely unable to provide enough care to consistently fledge all nestlings in the brood.

**Table 6.** Hatching success and number of nestlings lost for monogamous, primary, and secondary females.

	Hatching success			Nestlings lost		
Female status	Mean eggs hatched/laid	SE	*n* nests	Mean number of nestlings	SE	*n* nests
Monogamous	0.87	0.01	1,465	0.29	0.02	1,030
Primary	0.93	0.01	326	0.32	0.04	244
Secondary	0.87	0.01	341	0.38	0.04	260

Relatively few studies have investigated the consequences of polygyny for recruitment rates, and to our knowledge, the only other studies to find reduced recruitment of young secondary females in a facultatively polygynous passerine have been on the polyterritorial European pied flycatcher (*Ficedula hypoleuca*; Lubjuhn et al. 2000; Both 2002; Huk and Winkel 2006; but see Canal et al. 2021). Male care may be even more essential in the post-fledging stage than in the nestling stage due to brood division: after fledging, the parents divide the brood and each assumes primary responsibility for protecting and feeding 1 to 3 fledglings (Wheelwright et al. 1992, 2003). Polygynous males must divide their attention between young from multiple nests and often care preferentially for young from their primary nest (Alatalo et al. 1982; Smith et al. 1982; Huk and Winkel 2006); fledglings that receive less food provisioning and protection from males would likely be in worse condition or more likely to be depredated, and thus less likely to recruit.

When considered in combination with age, mating status also influenced the survival of females: yearling secondary females had lower survival (43%) than older secondary females (56%). Yearlings are less experienced breeders, and in the Kent Island population, have lower reproductive success than older females (Wheelwright and Schultz 1994). Young secondary females are potentially less able to bear the costs of raising young on their own compared to older, more experienced females, and thus may experience some tradeoff in survival versus reproductive effort. We also found significantly lower survival between older and yearling monogamous females. However, this is likely an artifact of the population structure: since there are more monogamous females, we have more observations of very old monogamous females that are probably more likely to die from old-age-related causes than younger females (MO: 18 records where females are 5+ yr old, out of 948 monogamous). On the other hand, we have no observations of very old secondary females, so among these, there are likely no old age-related deaths (PG 2: 3 records where females are 5+ yr old, and none 6 to 7 yr old, out of 275 secondary).

Unlike prior studies on several other species, our results do not provide support for the polygyny threshold model, which predicts that secondary females will not show reduced fitness relative to primary or monogamous females because they compensate for the costs of polygyny by choosing higher-quality breeding situations (Orians 1969; Temrin et al. 1996; Pribil and Searcy 2001; Garamszegi et al. 2004; Canal et al. 2021). In this study population of Savannah sparrows, as in many other species, females paid a cost in several indices of fitness by mating with an already-mated male, which was not compensated for by the increased quality of territories or mates (Alatalo et al. 1982; Slagsvold and Lifjeld 1994; Both 2002; Kujala et al. 2022). Studies that found no cost to secondary females may simply have overlooked such a cost by not investigating a wide enough range of fitness indices (Searcy and Yasukawa 1989). On the other hand, the cost of polygyny may be higher in some species and situations than in others: for instance, when environmental conditions are favorable, male help may be less necessary to successfully raise young, and polygyny may be less detrimental (Weatherhead 1979; Canal et al. 2021). On Kent Island, variation in territory quality among males may be limited in its extent or importance, especially because Savannah sparrows often forage in communal, off-territory areas such as the intertidal zone (Wheelwright et al. 1992; Wheelwright and Schultz 1994). Limited variation in territory quality would mean that even by choosing the “best-quality” territories, females cannot offset the costs of sharing a territory and a mate. The question remains, then, why is polygyny maintained in the Kent Island population despite the cost to secondary females?

Given the lack of support for the polygyny threshold model, our work suggests that secondary females are likely “making the best of a bad situation” (Schlicht and Kempenaers 2021). If no unmated males are available, females must choose between becoming the secondary female of an already-mated male or not breeding at all. This situation may be fairly common in the Kent Island population, where the sex ratio of breeders is consistently female-biased (Wheelwright et al. 1994, DRN, DJM, SMD, AEMN, unpublished data). Because Savannah sparrows attempt to breed every year (Wheelwright and Rising 2020) and even secondary females are still able to produce some recruiting offspring, it is unlikely that females would choose not to breed over mating polygynously. On Kent Island, polygynous matings typically arise in one of 3 situations (Wheelwright et al. 1992). First, young females, which usually arrive later than older females and thus have limited options, mate with already-mated males. Second, when a territorial male dies or leaves his territory in the middle of the breeding season (e.g. to follow fledglings from the first brood), a neighboring male may expand his territory and mate with the “widowed” resident female in addition to his original mate(s). Third, unbanded females occasionally appear several weeks into the breeding season, perhaps after a failed nest attempt elsewhere or to follow fledglings into the study area and may join already-mated males. In each of these situations, most males are already paired and females have little choice between mating monogamously or polygynously. Additionally, strong breeding philopatry in the Kent Island population (Wheelwright and Mauck 1998) means that females may have strong preferences for specific territories or spatial locations that outweigh avoidance of polygynous mating.

The other best-studied case of facultative polygyny in a passerine is that of the European pied flycatcher (Alatalo et al. 1981, 1982; Alatalo and Lundberg 1990; Lifield and Slagsvold 1990; Lubjuhn et al. 2000; Both 2002; Huk and Winkel 2006; Canal et al. 2021; Santoro et al. 2022), but there are vastly different spatial dynamics underlying the polygynous systems of this species and Savannah sparrows. Unlike Savannah sparrows, male pied flycatchers are polyterritorial, defending only a nest cavity as a territory, with multiple mates and nests on separate territories that can be as much as a few kilometers apart. In pied flycatchers, the “deception hypothesis” has been proposed to explain the evolution of polygyny, where males “deceive” females into mating with them by concealing that they are already mated (Alatalo et al. 1981, 1982). Polyterritoriality allows males to more effectively hide their mating status from potential mates because females are spatially separated (Alatalo et al. 1982). Savannah sparrow males hold only a single, relatively small territory in open habitat (~0.1–1 ha, Wheelwright and Rising 2020; on Kent Island, 0.006 to 0.6 ha, Suarez Sharma et al. 2024), so it is much less likely that male sparrows would be able to conceal their mating status from females. Additionally, the larger spatial separation between nests in polyterritorial species could heighten the disparity in male care and reproductive success between monogamous, primary, and secondary females by making it more difficult for males to attend multiple nests. However, even in our mono-territorial system, where it is potentially easier for males to simultaneously care for multiple broods, we still found differences in the success of females by mating status.

Our conclusions are based heavily on the assumption that males provide differential care to monogamous, primary, and secondary females. However, we did not actually measure rates of male parental care as we were limited to the data available in our long-term nest monitoring dataset. Males of many species have been found to provide less care at the nests of secondary females (Alatalo et al. 1981, 1982; Smith et al. 1982; Johnson et al. 1994; Moreno et al. 2002). Wheelwright et al. (1992) and Freeman-Gallant (1998) found no significant differences in Savannah sparrow males’ nestling provisioning rates at nests of different mating statuses; males did *tend* to provide less care at secondary nests, though this difference was not significant, potentially due to small sample sizes and high variability (polygynous: *n* = 12 broods, monogamous: *n* = 22 broods; sample sizes for primary vs. secondary not provided; Wheelwright et al. 1992). Additionally, these observations of feeding rates occurred across only a few years, and the short study period may not have captured sufficient environmental variation to reveal differences between monogamous and polygynous males (Weatherhead 1979; Canal et al. 2021). To our knowledge, no study has compared male feeding rates for monogamous, primary, and secondary broods during the fledgling period when male parental care may be particularly important (Wheelwright et al. 1992; Verhulst and Hut 1996), so the assumption that males provide less care to fledglings of secondary females remains untested. Other possible explanations for differences between secondary and primary/monogamous females: Secondary females are generally somewhat younger than primary or monogamous females (PG2: 1.57 ± 0.05 years, *n* = 408; PG1: 1.94 ± 0.05 years, *n* = 377; MO: 1.73 ± 0.02 years, *n* = 1,898), and, by definition, begin breeding later in the season, often due to later arrival from migration. We did find both age and date effects for most fitness indices; younger females were less successful in all cases, though the relationship was more complicated for date effects (e.g. number of fledglings per nest decreased with date, but fledging success was highest in the middle of the season). If age was the primary driver of differences in our fitness indices by mating status, we would have expected to see the same pattern (reduced fitness of secondary females relative to the others) in all measures, since age differences should be consistent. Additionally, primary females were somewhat older than monogamous females on average, so we might have expected to see significant differences between primary and monogamous females, which we did not observe. We also included age and date effects in all models (except for the female survival model, which had no date effect).

Here, we show that mating polygynously reduces the fitness of secondary females in terms of number of fledglings produced, reduced recruitment of those fledglings, and decreased survival following their first year of breeding, which we propose is due to reduced male parental care for broods of secondary females both during the nestling and fledgling periods. Further support could be provided by measuring rates of feeding by males to broods of monogamous, primary, and secondary females, particularly during the post-fledging period. Our study also demonstrates the value of detailed, long-term population monitoring data for understanding mating systems and life history. The larger sample sizes and many years of data associated with a long-term study allowed us to detect the effects of polygyny on female reproductive performance even when they are relatively small or variable, especially given the potential variation in costs of polygyny among years (Weatherhead 1979; Santoro et al. 2022). We also add weight to the argument that multiple indices of fitness are needed to accurately analyze the cost of polygyny to females, given that we found an effect in some but not all metrics we studied (Searcy and Yasukawa 1989; Garamszegi et al. 2004). In particular, we encourage more studies focused on differences in recruitment between females of different mating statuses among a wider variety of species.

## Supplementary Material

arae093_suppl_Supplementary_Appendix_1

arae093_suppl_Supplementary_Appendix_2

## Data Availability

Analyses reported in this article can be reproduced using the data provided by Mueller et al. (2024).
