# Supplementary material for: Reduced fitness of secondary females in a polygynous species: a 32-yr study of Savannah sparrows
Source: Behav Ecol. 2024 Nov 23;36(1):arae093. doi: 10.1093/beheco/arae093 (PMC11631185; doi:10.1093/beheco/arae093)
Supplement: arae093_suppl_Supplementary_Appendix_1 [file arae093_suppl_supplementary_appendix_1.docx]

**Supporting Information.** Mueller, S. D., N. T. Wheelwright, D. J. Mennill, A. E. M. Newman, S. M. Doucet, J. B. Burant, S. L. Dobney, G. W. Mitchell, H. A. Spina, B. K. Woodworth, and D. R. Norris. 2024. Reduced fitness of secondary females in a polygynous species: a 32-year study of Savannah sparrows. Behavioral Ecology.

**Appendix 1: Results of full models**

**Table S1.** ANOVA table (type III) for the full model for female survival in Savannah sparrows, with effects of female mating status; annual number of fledglings; population density; female age; the interactions of female mating status with number of fledglings, population density, and female age; the interactions of number of fledglings with population density and female age; and the interaction of population density with female age. Interactions with non-significant p-values (p > 0.05) were removed for the final model. Sample sizes: years = 27, unique females = 840, female-year combinations = 1,428.

| **Fixed effects** | **χ^2^ value** | **df** | ***p*-value** |
| --- | --- | --- | --- |
| (Intercept) | 15.95 | 1 | **<0.001** |
| Female status | 0.66 | 2 | 0.719 |
| Number of fledglings | 13.39 | 1 | **<0.001** |
| Population density | 0.00 | 1 | 0.995 |
| Female age | 1.13 | 1 | 0.287 |
| Female status × number of fledglings | 2.61 | 2 | 0.272 |
| Female status × population density | 1.22 | 2 | 0.544 |
| Female status × female age | 9.11 | 2 | **0.010** |
| Number of fledglings × population density | 0.03 | 1 | 0.863 |
| Number of fledglings × female age | 0.68 | 1 | 0.409 |
| Population density × female age | 0.98 | 1 | 0.322 |

**Table S2.** ANOVA table (type III) for the full model for clutch size in Savannah sparrows, with effects of female mating status; population density; female age; clutch number; first egg date; the interactions of female status with population density and clutch number; the interaction of population density and female age; and the interaction of clutch number and first egg day. Interactions with non-significant p-values (p > 0.05) were removed for the final model. Sample sizes: years = 30, unique females = 918, nests = 2,579.

| **Fixed effects** | **χ^2^ value** | **df** | ***p*-value** |
| --- | --- | --- | --- |
| (Intercept) | 1043.01 | 1 | **<0.001** |
| Female mating status | 4.39 | 2 | 0.112 |
| Population density | 0.06 | 1 | 0.809 |
| Female age | 49.59 | 1 | **<0.001** |
| Clutch number | 136.45 | 1 | **<0.001** |
| First egg day | 174.01 | 1 | **<0.001** |
| Female mating status × population density | 0.93 | 2 | 0.629 |
| Female mating status × clutch number | 1.73 | 2 | 0.422 |
| Population density × female age | 0.20 | 1 | 0.656 |
| Clutch number × first egg day | 111.45 | 1 | **<0.001** |

**Table S3.** ANOVA table (type III) for the full model for fledging success in Savannah sparrows, with effects of female mating status; brood size; female age; population density; the interactions of female mating status with brood size, female age, and population density; the interactions of brood size with female age and population density; and linear and quadratic effects of first egg day. Interactions with non-significant p-values (p > 0.05) were removed for the final model. Sample sizes: years = 27, unique females = 759, nests = 1,727.

| **Fixed effects** | **χ^2^ value** | **df** | ***p*-value** |
| --- | --- | --- | --- |
| (Intercept) | 99.23 | 1 | **<0.001** |
| Female mating status | 1.17 | 2 | 0.557 |
| Brood size | 1.77 | 3 | 0.622 |
| First egg day | 38.07 | 1 | **<0.001** |
| First egg day^2 | 4.48 | 1 | **0.034** |
| Female age | 6.22 | 1 | **0.013** |
| Population density | 0.42 | 1 | 0.518 |
| Female mating status × brood size | 6.18 | 6 | 0.403 |
| Female mating status × female age | 0.22 | 2 | 0.896 |
| Female mating status × population density | 4.14 | 2 | 0.126 |
| Brood size × female age | 7.35 | 3 | 0.061 |
| Brood size × population density | 6.01 | 3 | 0.111 |

**Table S4.** ANOVA table (type III) for the full model for fledglings produced per nest in Savannah sparrows, with effects of female mating status; female age; population density; first egg date; clutch number; the interactions of female status with female age, first egg date, population density, and clutch number; the interaction of female age with population density; and the interaction of first egg day with clutch number. Interactions with non-significant p-values (p > 0.05) were removed for the final model. Sample sizes: years = 30, unique females = 875, nests = 1,982.

| **Fixed effects** | **χ^2^ value** | **df** | ***p*-value** |
| --- | --- | --- | --- |
| (Intercept) | 8047.35 | 1 | **<0.001** |
| Female mating status | 6.80 | 2 | **0.033** |
| Female age | 4.04 | 1 | **0.044** |
| Population density | 0.17 | 1 | 0.684 |
| First egg day | 66.19 | 1 | **<0.001** |
| Clutch number | 64.00 | 1 | **<0.001** |
| Female mating status × female age | 0.14 | 2 | 0.934 |
| Female mating status × first egg day | 1.15 | 2 | 0.563 |
| Female mating status × population density | 0.29 | 2 | 0.866 |
| Female mating status × clutch number | 1.46 | 2 | 0.482 |
| Female age × population density | 0.03 | 1 | 0.865 |
| First egg day × clutch number | 14.64 | 1 | **<0.001** |

**Table S5.** ANOVA table (type III) for the full model for fledglings produced per year in Savannah sparrows, with effects of female mating status; number of successful nests; population density; breeding start date; female age; the interactions of female status with population density and female age; and the interaction of population density with female age. Interactions with non-significant p-values (p > 0.05) were removed for the final model. Sample sizes: years = 27, unique females = 611, female-year combinations = 987.

| **Fixed effects** | **χ^2^ value** | **df** | ***p*-value** |
| --- | --- | --- | --- |
| (Intercept) | 15205.05 | 1 | **<0.001** |
| Female mating status | 9.03 | 2 | **0.011** |
| Number of successful nests | 1893.70 | 1 | **<0.001** |
| Population density | 0.79 | 1 | 0.375 |
| Start date | 11.28 | 1 | **0.001** |
| Female age | 3.23 | 1 | 0.072 |
| Female mating status × population density | 2.21 | 2 | 0.332 |
| Female mating status × female age | 0.97 | 2 | 0.617 |
| Population density × female age | 0.19 | 1 | 0.664 |

**Table S6.** ANOVA table (type III) for the full model for recruitment of fledgling Savannah sparrows, with effects of mother’s mating status; population density; combined ages of parents; fledge date; mass as nestling; the interactions of mother’s mating status with population density and parents’ ages; and the interactions of population density with parents’ ages and fledge date. Interactions with non-significant p-values (p > 0.05) were removed for the final model. Sample sizes: years = 27, unique females = 706, fledglings = 5,172.

| **Fixed effects** | **χ^2^ value** | **df** | ***p*-value** |
| --- | --- | --- | --- |
| (Intercept) | 551.93 | 1 | **<0.001** |
| Mother’s mating status | 5.17 | 2 | 0.075 |
| Population density | 1.19 | 1 | 0.275 |
| Parents’ ages | 5.50 | 2 | 0.064 |
| Fledge day | 27.40 | 1 | **<0.001** |
| Mass | 16.38 | 1 | **<0.001** |
| Mother’s mating status × population density | 0.88 | 2 | 0.643 |
| Mother’s mating status × parents’ ages | 7.54 | 4 | 0.110 |
| Population density × parents’ ages | 2.73 | 2 | 0.256 |
| Population density × fledge day | 0.73 | 1 | 0.392 |
