# Supplementary material for: Reduced fitness of secondary females in a polygynous species: a 32-yr study of Savannah sparrows
Source: Behav Ecol. 2024 Nov 23;36(1):arae093. doi: 10.1093/beheco/arae093 (PMC11631185; doi:10.1093/beheco/arae093)
Supplement: arae093_suppl_Supplementary_Appendix_2 [file arae093_suppl_supplementary_appendix_2.docx]

**Supporting Information.** Mueller, S. D., N. T. Wheelwright, D. J. Mennill, A. E. M. Newman, S. M. Doucet, J. B. Burant, S. L. Dobney, G. W. Mitchell, H. A. Spina, B. K. Woodworth, and D. R. Norris. 2024. Reduced fitness of secondary females in a polygynous species: a 32-year study of Savannah sparrows. Behavioral Ecology.

**Appendix 2**

Table S1. The effects of female mating status, population density, female age, clutch number, first egg date, and the interaction of clutch number and first egg date on clutch size in Savannah sparrows.

| **Fixed effects** | **Estimate (β)** | **SE** | **95%CI** | ***z*-value** | ***p*-value** |
| --- | --- | --- | --- | --- | --- |
| (Intercept) | -0.227 | 0.007 | (-0.241, -0.214) | -33.01 | <0.001 |
| Female status MO^1^ | 0.001 | 0.004 | (-0.007, 0.009) | 0.36 | 0.722 |
| Female status PG1^2^ | 0.008 | 0.005 | (-0.002, 0.018) | 1.50 | 0.135 |
| Population density | 0.000 | 0.003 | (-0.007, 0.006) | -0.09 | 0.929 |
| Female age ASY^3^ | 0.019 | 0.003 | (0.013, 0.024) | 7.01 | <0.001 |
| Clutch number 1 | 0.048 | 0.003 | (0.041, 0.054) | 14.61 | <0.001 |
| First egg day | -0.006 | 0.000 | (-0.007, -0.005) | -13.18 | <0.001 |
| Clutch number × first egg day | 0.004 | 0.000 | (0.004, 0.005) | 10.54 | <0.001 |
| **Random effects** | **Variance (σ^2^)** | **SD** |  |  |  |
| Year | 0.001 | 0.028 |  |  |  |
| Female ID | 0.004 | 0.061 |  |  |  |

Abbreviations: ^1^Monogamous; ^2^Primary polygynous; ^3^After second-year

Notes: Values in bold indicate significant differences. Sample sizes: years = 30, unique females = 918, nests = 2,579.

Table S2. The effects of female mating status, brood size, female age, population density, and linear and quadratic effects of first egg day on fledging success in Savannah sparrows.

| **Fixed effects** | **Estimate (β)** | **SE** | **95%CI** | ***z*-value** | ***p*-value** |
| --- | --- | --- | --- | --- | --- |
| (Intercept) | 2.242 | 0.208 | (1.833, 2.650) | 10.76 | <0.001 |
| Female status MO^1^ | -0.038 | 0.122 | (-0.277, 0.200) | -0.31 | 0.753 |
| Female status PG1^2^ | -0.201 | 0.153 | (-0.501, 0.099) | -1.31 | 0.189 |
| Brood size = 1-2 nestlings | -0.555 | 0.245 | (-1.035, -0.074) | -2.26 | 0.024 |
| Brood size = 4 nestlings | 0.357 | 0.132 | (0.098, 0.617) | 2.70 | 0.007 |
| Brood size = 5 nestlings | 0.019 | 0.164 | (-0.302, 0.340) | 0.12 | 0.908 |
| First egg day | 0.038 | 0.006 | (0.025, 0.050) | 5.99 | <0.001 |
| First egg day^2 | -0.001 | 0.000 | (-0.002, 0.000) | -2.17 | 0.030 |
| Female age ASY^3^ | 0.206 | 0.083 | (0.044, 0.369) | 2.49 | 0.013 |
| Population density | 0.104 | 0.063 | (-0.019, 0.228) | 1.65 | 0.098 |
| **Random effects** | **Variance (σ^2^)** | **SD** |  |  |  |
| Year | 0.184 | 0.429 |  |  |  |
| Female ID | 0.465 | 0.682 |  |  |  |

Abbreviations: ^1^Monogamous; ^2^Primary polygynous; ^3^After second-year

Notes: Values in bold indicate significant differences. Sample sizes: years = 27, unique females = 759, nests = 1,727.
